# Behavioral and cognitive changes after early postnatal lesions of the rat mediodorsal thalamus

**DOI:** 10.1016/j.bbr.2015.06.017

**Published:** 2015-10-01

**Authors:** Zakaria Ouhaz, Saadia Ba-M’hamed, Anna S. Mitchell, Abdeslem Elidrissi, Mohamed Bennis

**Affiliations:** a Laboratory of Pharmacology, Neurobiology and Behavior (URAC-37), Cadi Ayyad University, Marrakech, Morocco; b Department of Experimental Psychology, University of Oxford, South Parks Road, Oxford OX1 3UD, United Kingdom; c Biology Department, College of Staten Island, The City University of New York, Staten Island, USA

**Keywords:** MD, mediodorsal nucleus of the thalamus, PFC, prefrontal cortex, ZT, Zeitgeber time, P4, postnatal day 4, AP, antero-posteriority, L, laterality, P, profoundness, US, unconditioned stimulus, CS, conditioned stimulus, R, Ratio, ATN, anterior thalamic nucleus, VTA, ventral tegmental area, SA, spontaneous activity, Lesion, Mediodorsal thalamus, Anxiety, Learning, Prefrontal cortex, Schizophrenia

## Abstract

Early insults to the thalamus result in functional and/or structural abnormalities in the cerebral cortex. However, differences in behavioral and cognitive changes after early insult are not well characterized. The present study assessed whether early postnatal damage to mediodorsal nucleus of the thalamus (MD), reciprocally interconnected with the prefrontal cortex, causes behavioral and cognitive alterations in young adult rats. Rat pups at postnatal day 4 received bilateral electrolytic lesion of MD, or a MD Sham lesion or were anesthetized controls; on recovery they were returned to their mothers until weaning. Seven weeks later, all rats were tested with the following behavioral and cognitive paradigms: T-maze test, open field test, actimetry, elevated plus maze test, social interactions test and passive avoidance test. Rats with bilateral MD damage presented with disrupted recognition memory, deficits in shifting response rules, significant hypoactivity, increased anxiety-like behavior, deficits in learning associations as well as decreased locomotor activity, and reduced social interactions compared to MD Sham lesion and anesthetized Control rats. The lesion also caused significant decreases in pyramidal cell density in three frontal cortex regions: medial infralimbic cortex, dorsolateral anterior cortex, and cingulate Cg1 cortex. The present findings suggest a functional role for MD in the postnatal maturation of affective behavior. Further some of the behavioral and cognitive alterations observed in these young adult rats after early MD lesion are reminiscent of those present in major psycho-affective disorders, such as schizophrenia in humans.

## Highlights

• Early insult of the mediodorsal thalamus (MD) disturbed cognitive behaviors.
• Early MD damage decreased locomotor activity, and reduced social interactions.
• Early insult of the MD disturbed postnatal maturation of affective behavior.
• The MD is important for prefrontal cortex function during brain maturation.

## 1 Introduction

There is increasing interest in modeling some of the cognitive deficits in schizophrenia with paradigms that are suitable for both humans and experimental animals. These cognitive deficits include impairments in working memory and behavioral flexibility [1,2]. Altered activity in the prefrontal cortex (PFC) has been associated with these cognitive symptoms [3–5]. However, the cognitive symptoms of schizophrenia, typically linked to prefrontal cortex (PFC) dysfunction remain essentially resistant to treatment [6]. Thus, it remains critical to understand the underlying neural mechanisms of these symptoms in order to target effective treatments, because cognitive symptoms are highly predictive for the long-term prognosis of the disease [7,8].

Recently, the mediodorsal thalamus (MD) has become a focus of attention in the study of cognitive symptoms and schizophrenia, mainly due to its dense excitatory reciprocal connections with the PFC [9]. Neuroimaging and postmortem anatomy in schizophrenic patients indicate that MD is shrunken with neuronal loss, or has metabolic changes [10,11]. Behavioral neuroscience studies using monkeys or rodents have indicated that MD contributes to learning, memory, decision-making, fear conditioning, anxiety, and impaired social interactions [8,12–20]. Further early postnatal unilateral electrolytic lesions of MD at postnatal day 4 disrupt dendritic development of PFC pyramidal cells [21]. This evidence from animal models suggests that changes to MD observed in patients with schizophrenia may be associated with some of the cognitive symptoms associated with this neuropsychiatric disorder.

Given that schizophrenia is a developmental disorder, traditionally associated with dysfunction in the PFC, the longer-term cognitive and behavioral effects of neonatal lesions within the medial PFC have been studied in rodent models. Interestingly, rats with PFC lesions induced at postnatal day 7 have deficits in cognitive control-like behaviors [22], but intact working memory, suggesting that early developmental damage to the PFC can lead to impaired executive functioning but does not impact on memory. Thus far, after early onset damage to the MD, adult rats display reduced vertical activity in an open field test [23] but learning remained intact using an operant delayed alternation task [24]. However, further assessments of changes in their affective state and cognition have not been conducted.

Thus, the present study tested the longer-term cognitive and behavioral effects of early developmental insult to MD in adult rats. A battery of cognitive and behavioral tasks assessed the generality of social interactions and anxiety, as well as learning and memory. We hypothesized that if the influence of the MD is critical for the normal, ongoing development of the PFC from the earliest postnatal stages of brain maturation then MD surgical lesions induced just after birth should cause long-term alterations in cognition and behavior in adult rats.

## 2 Material and methods

### 2.1 Animals

The experiments involved 67 Sprague–Dawley rats, bred in the central animal care facilities of Cadi Ayyad University, Marrakech, Morocco. During the training sessions, five animals were excluded from the study, because they showed a high level of anxiety (freezing); thus, the final number was 60 rats (47 males/13 females; sex was counter balanced between groups). After birth, the rats (weighing 6 g ±2) were housed with their mothers in litters and kept under constant temperature conditions (20 °C ±2), using a 12 h light/12 h dark cycle (lights on at 7 am: ZT 0), with water and food ad libitum. Among these 60 rats, 18 rats were used for the delayed alternation tasks, actimetry, and for the quantification of the PFC cells density, while the remaining 42 rats were used for the assessments of the other behavioral tasks described below.

The study received approval of the Council Committee of research laboratories of the Faculty of Sciences, Cadi Ayyad University of Marrakech. All procedures were conducted in accordance with the approved institutional protocols and within the provisions for animal care and use prescribed in the scientific procedures on living animals, European Council Directive (EU2010/63). All efforts were made to minimize any animal suffering.

### 2.2 Surgery

Previous brain developmental studies have documented that in rodents, MD afferents reach the PFC between postnatal days 1–7, while PFC efferents reach the MD during postnatal days 4–9 [25]. Thus the MD lesions were performed on postnatal day 4 (P4) in order to alter the communication between the MD and PFC during this critical period of postnatal brain development. Date of birth was designated as postnatal day P0. The animals were randomly divided into three groups (*n* = 19 per group): group 1, pups received a bilateral electrolytic lesion in MD; group 2, MD Sham lesion (electrode placement only); group 3, naïve (unlesioned) but anesthetized Controls. On P4, all pups were anesthetized by hypothermia for 5 min [23] and placed in an adapted stereotactic apparatus using aseptic conditions. In groups 1 and 2, a skin incision was made, just lateral to the midline (to avoid the mid-sagittal sinus), their scalp was then retracted, and the skull, bregma and the intended lesion site were gently exposed. The electrode was inserted through the skull, which is still very soft at day P4 using the following coordinates from bregma: AP = −1 mm, *L* = ±0.5, and *P* = −3.5 mm. (These coordinates had been previously determined in pilot neurosurgeries in a different cohort of P4 rats in which the electrode track placement was histologically verified using thionin blue-stained serial sections). In the animals of group 1 only, a current of 5 mA (tip negative) was then passed for 3 s. In groups 1 and 2, the electrode was removed, and then the skin was repositioned and held in place with super-glue (ARADINE, Morocco). After surgery, the wounds were cleaned by betadine 10% (Pharma Laboratory, Morocco) and the pups were warmed up and returned to their mothers, where they remained until weaning at postnatal day 30. During the post-weaning period, groups of 5 rats were housed together per cage (40 cm × 25 cm × 18 cm). Seven weeks post-surgery (P53), animals were trained in the behavioral paradigms and assessments were made for any changes to their behavior and cognition.

### 2.3 Histology

After the behavioral testing was completed, the animals (*n* = 60) were deeply anesthetized with urethane 40% (1 g/kg, from Sigma–Aldrich, France), and then transcardially perfused with saline (0.9%), followed by 4% paraformaldehyde in phosphate-buffered saline (0.2 M). The brains were extracted from the skull and postfixed in the fixation solution for 12h, cryoprotected overnight in 30% sucrose, and then sectioned in coronal plane at a 40 μm thickness using a cryostat (Leica Microsystems, Germany). Serial sections through the thalamus were Nissl-stained using thionin blue.

### 2.4 Cell density measurements

In the rat, the MD projects to medial prelimbic cortex, dorsolateral anterior cortex and cingulate Cg1 cortex. The slides containing these areas of interest were determined using cresyl violet stained sections based on gross anatomical boundaries according to Faul and Mehler [26]. We referred to Paxinos et al. [27] to locate the gross borders of medial prelimbic cortex. To distinguish the cytoarchitectural borders of dorsolateral anterior cortex and cingulate cortices we used criteria from Gabbott et al. [28]. All of the sections containing the prefrontal regions were analyzed for cell density. Cells were counted in three evenly spaced sections with a random start. Contours were traced around each region of interest and a counting grid superimposed on the contour. Every third intersection with a random start was marked for counting. A counting box of 100 × 100 μm (with a buffer zone of 5 μm corresponding to the exclusion line on either surface was employed to exclude the cut surfaces) was centered on the marked intersections. Counting was done using a 40× Plan Apo objective under immersion oil. Every cell inside the box was counted that had a visible nucleolus that did not touch the exclusion line. Neuron density was determined by dividing the average number of cells per box by the surface of the counting box.

### 2.5 Behavioral testing

Prior to any behavioral testing, all of the animals were gently handled for 2 min/day during 1 week prior to the start of training. In addition, they were individually familiarized with the testing room and the test arena for 5 min/day during the daily handling prior to training. All training and testing was conducted in the same experimental testing room except for the passive avoidance test, which was conducted in an adjacent room. On each of the test days, rats were transported in their home cages to the testing room, and allowed between 20 and 40 min to acclimatize prior to testing.

Behavioral testing was conducted during sessions in daytime between ZT2 and ZT5 each except for the passive avoidance test, which was performed between ZT6 and ZT11, and the experimenter was blind to the surgical lesions of the animals. All animals (*n* = 42) from the three groups were tested (*n* = 5 or 6 per session) while their behavior was recorded and analyzed using Ethovision XT Noldus 8.5 video tracking program (Noldus Information Technology b.v., Wageningen, The Netherlands) connected to a video camera (JVC). The video camera was positioned 2.5 m above the arena, inside the vertical projection of a wall, covering the entire view of the arena. Tracking of the animal was based on contrast relative to background. Two tracking points were specified: one the head and the other the center of gravity of the animal.

#### 2.5.1 Delayed alternation test

This test was performed to investigate the contributions of the PFC and MD, to spatial working memory and inhibitory control processes, and to demonstrate the functional disturbance of the PFC after early MD lesion.

##### 2.5.1.1 T-maze non matching-to-place

On each day of training, the rats (control: *n* = 6; MD sham: *n* = 6; MD lesion *n* = 6) were transported from the vivarium to the laboratory, and training began 60 min later. The rats were initially habituated to a T-maze (dimensions, 90 × 80 cm) for 7 days until they were readily eating chocolate chips placed in food wells at the end of each arm. After habituation, the rats were trained on a delayed alternation task described previously by [29–31]. Rats were placed in the start box of the maze, the gate was opened, and the animal was allowed to run to a choice point in the maze. On the “sample run”, food was placed at the ends of both arms and the rat was rewarded for entering either arm. Then the rat was placed back in the start box for the intertrial delay. On the subsequent trial, the “choice run”, the rat was rewarded only for entering the arm not chosen in the previous trial. If the correct choice was made, the rat was rewarded and then placed back in the starting box for the intertrial delay. If the incorrect arm was chosen, the rat was placed in the start box without the reward. Between each trial, the choice point was wiped clean with 70% ethanol to remove any olfactory cues. Each day consisted of 11 trials, although the first trial was not included in the analysis. Accuracy of response and response time were scored, with response time being the time from when the start gate was opened to when the animal made its choice and reached one food well. Rats were tested once daily, five times per week. The intertrial delay was set at “2” second and then increased in 10 s increments as needed to stabilize performance at ≈80% accuracy.

##### 2.5.1.2 T-maze delayed matching-to-place

The habituation procedures were the same as those employed in the non matching-to-place experiment. During the testing period, each trial was divided into two stages a “sample run” preceded in an identical manner as in non matching-to-place procedure. However, during the “choice run” the animal was now required to match-to-place, thus if the rat ran down the same arm it had previously visited during the “sample run” it was allowed to eat the food reward and was then returned to the home cage. Conversely, if the other arm was chosen, the animal was confined to that arm for 10 s, and then returned to the home cage. All rats were tested in groups of 5, with each rat having one trial in turn. Each animal received a single session of 6 trials per day, with an intertrial delay of 5–6 min (the sequence consisted of three correct right and three correct left choices between the two arms) until a criterion level of five out of six correct trials on five consecutive sessions was reached. The total number of errors that were made before reaching criterion was recorded.

All the animals were perfused after these experiments and the actimetry to verify the lesion site and to perform the quantification of cell density in the PFC.

#### 2.5.2 Open field test

This test is widely used to assess locomotor activity and emotional reactivity in rodents (e.g., [32]). Locomotor activity and thigmotaxis were assessed during a 10 min session (control: *n* = 14; MD sham: *n* = 14; MD lesion: *n* = 14). The open field apparatus used was a 100 × 100 cm square arena with 30 cm high black walls. The animal was placed in the center of the arena and was covered by a small dome that was then pulled up by the operator at the beginning of video recording of their activity. After 10 min, the animal was returned to its home cage, and the apparatus cleaned with a damp sponge to remove any trace of odor.

#### 2.5.3 Assessment of spontaneous locomotor activity

For monitoring locomotor activity, rats (control: *n* = 6; MD sham: *n* = 6; MD lesion: *n* = 6) were housed individually in cages equipped with passive infrared motion captors, placed over the cages and a computerized data acquisition system (Circadian Activity Monitoring System). Activity records were analyzed with the Clocklab software package (Actimetrics, Evanston, IL). Total locomotor activity was recorded in a 12 h light:12 h dark cycle (12 L:12 D). The total behavioral recording period lasted 40 days, recording started from P53.

#### 2.5.4 Elevated-plus-maze test

The elevated plus maze is a widely accepted paradigm used to assess anxiety-like behavior in rodents [33–35]. The elevated plus-maze comprised two opposing open arms (50 × 5 cm) and two closed arms (50 × 5 × 15 cm), which joined at a square central area (5 × 5 cm) to form a plus sign. The maze floor and the side/end walls (15 cm height) of the enclosed arms were made of clear Plexiglas. To reduce the likelihood of falling from the arms, a slight raised edge (0.25 cm) around the perimeter of the open arms provided additional grip for the rats. The entire apparatus was elevated to a height of 45 cm above the floor. For testing, the rats (control: *n* = 14; MD sham: *n* = 14; MD lesion: *n* = 14) were removed gently from their home cages and placed in the central arena of the elevated-plus-maze, facing the junction of an open and closed arm. The experimenter retired behind a screen to shield them from the rats’ view. The rat was allowed to freely explore the maze for 5 min, while their behavior was recorded for offline analysis using a camera mounted to the ceiling, or on a frame above the apparatus. After testing each rat, any debris (e.g., bedding and faeces) was removed from the maze, and all interior surfaces, walls and floors were cleaned thoroughly, first with 70% ethanol, then water, and then wiped dry. At least 5 min was allowed between testing each individual animal to ensure the maze was completely dry and any residual odor of alcohol had dispersed before the next rat was introduced into the maze.

#### 2.5.5 Social contact test

This test was employed to validate the results obtained from the elevated plus maze and the open field test, allowing further assessments of social behavior and the development of any antisocial behavior caused by the MD lesion. Animals (control: *n* = 14; MD sham: *n* = 14; MD lesion: *n* = 14) were housed individually in their home cages for 24h prior to behavioral testing. During this time, their cages were positioned very close together in order to maintain auditory and olfactory contact. The test was performed in an open field test arena (100 × 100 × 35 cm), with lighting conditions similar to those of the animal facility. At the beginning of the social contact test, the experimental and stimulus rats were placed at opposite corners of the arena facing away from each other. The two animals were differentiated by marking the backs or tails of the stimulus rats with an indelible marker, with a color reserved solely for stimulus rats. The two rats in each pair were matched for age, gender and weight. During the test, each experimental animal was paired with a ‘neutral’ stimulus animal (MD Sham *with* Control; MD lesion *with* Control, and MD lesion *with* MD Sham).

The amount of social contact behavior was measured in accordance with a previously published protocol [35]. Counts were made of the total number of escapes and approaches between the two animals during the total testing period (10 min). The dependent measure was the ratio (*R*) between weighted movement from (used as an objective measure for the intensity of avoidance) and weighted movement to (used as an objective measure for the intensity of approach). An approach was defined as the nose-point of the experimental animal in proximity relative to the body points of the stimulus animal, with the experimental animal considered to be exploring or sniffing the other animal.

#### 2.5.6 Passive avoidance test

The passive avoidance test is widely used to assess learning and memory of a mildly aversive stimulus (footshock) delivered in a particular place. Higher time ratios (and arm entry ratios) are suggestive of more anxiety-like behavior [36]. The test was carried out during the light phase (ZT6-11), and each animal (control: *n* = 14; MD sham: *n* = 14; MD lesion: *n* = 14) was housed individually during the test. The testing box apparatus had bright and dark compartments with a guillotine door in between. The delivery of electric shocks (0.5 mA for 2 s), the raising and lowering of the door and the latencies at which the animals stepped into the dark from the bright compartment were controlled. The training consisted of two sessions; during the first session rats were placed in the shock chamber for 2 min (habituation session), followed by a single trial in which the rats were placed on the bright compartment facing away from the door and allowed to enter the dark chamber. On the next training session, three such trials were given with an intertrial interval of 2 min (training session). The next day, the testing sessions started (for 8 separate testing sessions, with each session repeating the same protocol). During the 8 testing sessions, each animal was gently placed in the light compartment for 10 s, after which the guillotine door was raised, and the time the animal waited before crossing to the dark (shock) compartment was recorded as the latency. Once the animal crossed with all four paws to the dark compartment, the door was closed and a 0.5 mA foot shock (Unconditioned stimulus (US)) delivered for 2 s. The trial ended when the animal waited more than 360 s to cross to the dark side, or if it received an electrical shock in the dark side after crossing (Conditioned stimulus (CS)). During this test, two parameters were assessed: the testing session daily latencies and the total number of (US–CS) association.

### 2.6 Data analysis

Statistical analysis was performed using SigmaPlot 11.0 software. Inter-group comparisons were performed using two way analysis of variance (ANOVA) or one way ANOVA or non-parametric Independent Samples Kruskal–Wallis ANOVA, followed by post-hoc testing with the Holm–Sidak method for pairwise multiple comparisons. The significance threshold was set at *p *< 0.05.

## 3 Results

### 3.1 MD thalamic lesions

**Fig. 1:**
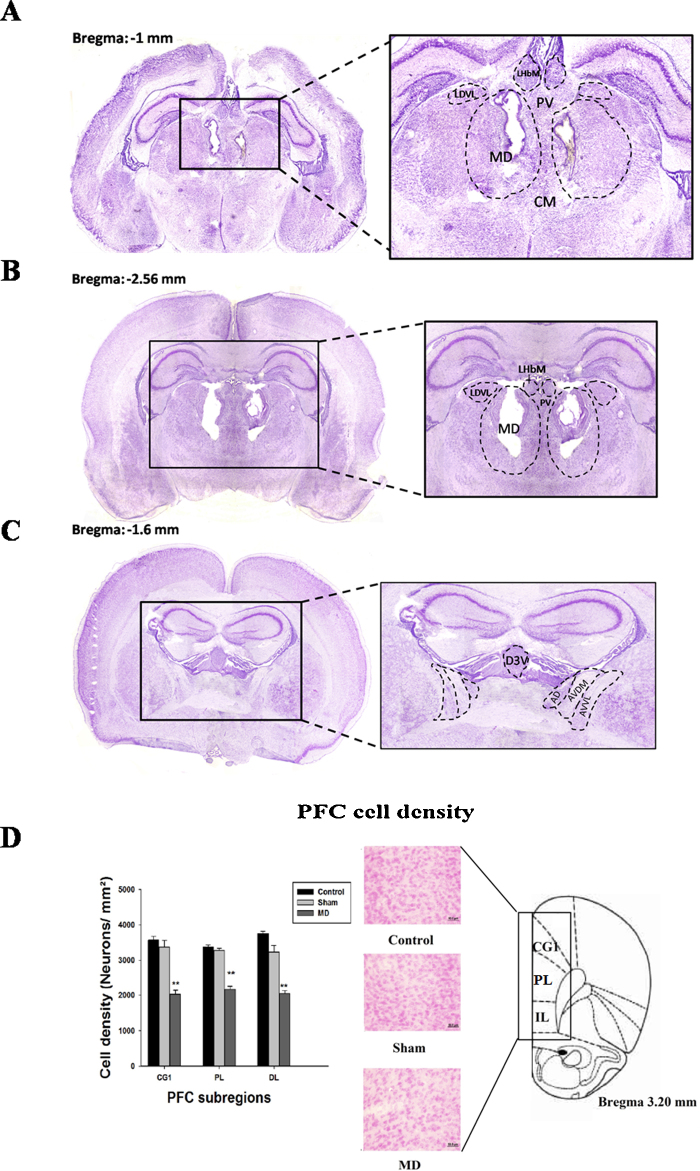
Photomicrographs detailing the extent of the damage to the MD at P4 (A), in adulthood rats which had bilateral lesions performed at P4 (B), and also the extent of the damage to the MD and to the ATN in adult rats (C). Numbers represent distance from bregma. (D) Assessment of early MD lesion at postnatal day 4 (P4) effect on pyramidal cells development in the prefrontal cortex. Abbreviations: CG1 = cingulate area 1 cortex; PL = prelimbic cortex; IL = infralimbic cortex. ** and ##: *p* < 0.01. The “*” refers to the control vs MD lesion group comparison and the “#” refers to the MD sham vs MD lesion group comparison.

The outcome of the electrolytic lesion performed at P4 (Fig. 1A) showed that all 20 adult rats (from the two sets of animals) had extensive lesions within MD (Fig. 1B). In all cases the lesion affected at least 90% of the nucleus, sparing only its most lateral and ventral portions. The MD region was consistently shrunken, and at the lesion site there was almost a complete loss of neurons. Limited damage was also found in the anterior dorsal thalamic nucleus in some cases (10/20) bilaterally and in three cases unilaterally (Fig. 1C). In all cases the other anterior thalamic nuclei were spared, but there was damage to the thalamic paraventricular nucleus at the midline. In most cases (18/20) there was also a limited area of damage in the habenulae. The histology also revealed some neuronal changes in the hippocampi of the MD lesion rats that were not apparent in the Sham MD or anesthetized Control groups (see Fig. 1A).

### 3.2 Cell density

**Fig. 2:**
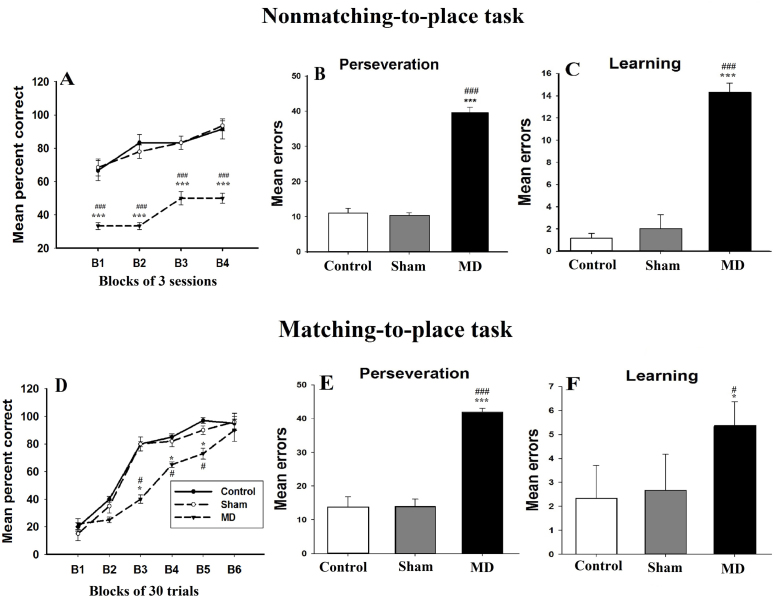
Acquisition of the delayed alternation task in T-maze. (A–C): animals performance in a nonmatching-to-place task. (A) The pattern of acquisition of the T-maze nonmatching-to-place over four blocks of three sessions. (B–C) Mean (±SD) group error scores have been divided into two phases: Perseveration phase (B) in which rats are performing below chance and learning phase (C) in which rats are performing at or above chance. (D–F) Acquisition of the delayed alternation responses in T-maze matching- to-place task caused by MD lesion at postnatal day 4 (P4). (D) The pattern of acquisition of the T-maze matching-to-place over six blocks of 30 trials sessions. (E and F) Mean (±SD) group error scores have been divided into two phases: Perseveration phase (E) in which rats are performing below chance; learning phase (F) in which rats are performing at or above chance. * and #: *p* < 0.05; ***: *p* < 0.001. The “*” refers to the control vs MD lesion group comparison and the “#” refers to the MD sham vs MD lesion group comparison.

To determine if the MD lesion caused retrograde cell loss, we examined pyramidal cell density in each of the three PFC regions (Fig. 1D). Our results showed that the MD lesion caused a decrease of cells density in the PFC. The two way repeated measures ANOVA (for two factors: lesion status and PFC regions) confirmed this findings [*F*(3,53) = 909.58, *p *< 0.001]. In the prelimbic region, the post-hoc analysis showed a significant difference between control (3376.66 cell/mm^3^ ± 56.43) and MD lesion groups (2171.16 ± 89.1) (*t* = 24.38, *p* < 0.001), between MD sham (3278.38 cell/mm^3^ ± 61.47; Fig. 2) and MD lesion groups (*t* = 21.06, *p* < 0.001), but did not show a significant difference between control and MD sham groups (*t* = 0.74, *p* > 0.05). In the dorsolateral region, the post-hoc analysis showed a significant difference between control (3756.66 cell/mm^3^ ± 62.05) and MD lesion groups (2048.33 cell/mm^3^ ± 84.59) (*t* = 29.94, *p* < 0.001), between MD sham (3235.33 cell/mm^3^ ± 181.59; Fig. 2) and MD lesion groups (*t* = 17.31, *p* < 0.001), but did not show a significant difference between control and MD sham groups (*t* = 0.49, *p* > 0.05). In the third region, cingulate cortex, the post-hoc analysis showed a significant difference between control (3570 cell/mm^3^ ± 104.40) and MD lesion groups (2030 ± 110.22) (*t* = 28.08, *p* < 0.001), between MD sham (3376.22 cell/mm^3^ ± 190.62; Fig. 1B) and MD lesion groups (*t* = 23.41, *p* < 0.001), but did not show a significant difference between control and MD sham groups (*t* = 1.69, *p* > 0.05).

### 3.3 Behavioral testing

#### 3.3.1 The delayed alternation task

In addition to examining overall trials and errors to criterion, acquisition was subdivided into two main phases. The first, “perseveration” corresponded to when rats were performing appreciably below chance (<3 out of 12, *p* = 0.073 binomial distribution), i.e., when the rats initially tried to solve the matching task by nonmatching-to-place. The second phase, “learning”, corresponded to when performance was at or above chance. To perform this analysis, we counted the number of correct responses made by each rat using a running window of 12 trials, beginning with trials 1–2 and advancing the window by one trial at a time. The initial perseveration phase was deemed to have ended when the rat achieved a score of four or more correct responses in a window of 12 trials. The “learning“phase comprised all subsequent trials up to the task acquisition criterion of five out of six correct trials on five consecutive sessions. An additional behavioral strategy was also noted during acquisition of the matching-to-place task. Having overcome the tendency to perseverate with a nonmatching strategy, rats frequently adopted a side bias (e.g., always turn right) before finally acquiring the matching task. This ‘side bias’ was examined by calculating both the percentage of sessions comprising the “learning” phase of matching-to-place acquisition in which the animal turned five times or more out of six in the same direction (left), and the absolute number of such sessions.

##### 3.3.1.1 Nonmatching-to-place

*Acquisition*: During the initial 12 acquisition sessions, each animal performed a total of 72 trials with a retention delay (2–5 s). The scores were grouped into four blocks, each of three sessions, and the mean percentage correct scores for each of the three groups are shown in (Fig. 2A). During the different test blocks, the pattern of acquisition was different between groups, the mean percentage correct scores of MD sham group were comparable to control group, and in contrast MD lesion animals were markedly impaired in their acquisition of the task with respect to both control and MD sham groups. Even by the end of the test (i.e., last block), the control group performed a median of 83.33% correct scores, the MD sham performed 83.16% of correct scores, but the MD lesion group performed below 80% accuracy criterion (41.66% of correct scores). The Kruskal–Wallis one way ANOVA for block four confirmed these differences [*H*(2) = 16.10, *p* < 0.001]. The post-hoc analysis showed a significant difference between control and MD lesion groups (*q* = 5.2, *p* < 0.05) and between MD sham and MD lesion groups (*q* = 6.53, *p* < 0.05), but did not show a significant difference between control and MD sham groups (*q* = 1.18, *p* > 0.05). The analysis of all test blocks using two way ANOVA test revealed a significant group effect [*F*(2,59) = 86.23, *p* < 0.001], a significant effect of session blocks [*F*(3,59) = 348, *p* < 0.001], and the group × session interaction was also significant [*F*(6,59) = 248.42, *p* < 0.001]. As described above, task acquisition was subdivided into two phases comprising “Perseveration” and “Learning”. During perseveration phase, the MD lesion group showed a highly perseverative behavior, they made more errors (39.6 ± 1.51) compared to MD sham (10.20 ± 0.83) and to control groups (10.60 ± 1.67; Fig. 2B). The one way ANOVA, confirmed these differences [*F*(2,14) = 735.13, *p* < 0.001]. The post-hoc analysis showed a significant difference between control and MD lesion groups (*t* = 32.97, *p* < 0.001) and between MD sham and MD lesion groups (*t* = 33.43, *p* < 0.001), but did not show a significant difference between control and MD sham groups (*t* = 0.45, *p* > 0.05). During learning phase, the MD lesion group made more errors (14.33 ± 0.81) compared to MD sham (2 ± 1.26) and to control groups (1.16 ± 0.4; Fig. 2C). The one way ANOVA, confirmed these differences [*F*(2.17) = 402.12, *p* < 0.001]. The post-hoc analysis showed a significant difference between control and MD lesion groups (*t* = 25.32, *p* < 0.001) and between MD sham and MD lesion groups (*t* = 23.71, *p* < 0.001), but did not show a significant difference between control and MD sham groups (*t* = 1.60, *p* > 0.05).

##### 3.3.1.2 T-maze matching-to-place

*Acquisition*: During the initial two blocks of 30 trials, as expected, animals from the three groups performed well below chance (Fig. 2), controls (22 ± 3%), MD sham (16.2 ± 5%) and MD lesion animals (18 ± 4%; Fig. 2D). The one way ANOVA confirmed that all groups performance at this stage was not significantly different from each other [*F*(2,17) = 4.02, *p* > 0.05]. At the third block, control (80 ± 2%) and MD sham groups started performing beyond chance and at 80% accuracy level (80 ± 5%; Fig. 2D). In contrast, the MD lesion group also performed beyond chance but did not reach the criterion (40 ± 3%). The one way ANOVA confirmed this difference [*F*(2,14) = 210.52, *p* < 0.001]. The post-hoc analysis showed that during the third block the difference between control and MD lesion group (*t* = 18.21, *p* < 0.001) and between MD sham and MD lesion group (*t* = 17.77, *p* < 0.001) are significant, but did not show any difference between control and MD sham groups (*t* = 0.44, *p* > 0.05). By the end of the test, MD lesion animals started performing beyond the criterion and they scored (90% ± 8) of correct choices which is statistically comparable to controls (95 ± 5%) and MD sham animals (96 ± 6.2%) [*F*(2,14) = 1.21, *p* > 0.05]. The analysis of the different test blocks using repeated measures ANOVA, showed a significant group effect [*F*(3,95) = 42.91, *p* < 0.001], and a significant effect of session blocks [*F*(5,95) = 243.92, *p* < 0.001], but the group × session interaction was not significant [*F*(15,95) = 5.2, *p *> 0.05].

During perseveration phase, the MD lesion group showed a highly perseverative behavior, they made more errors (42 ± 2.55) compared to MD sham (14 ± 2.12) and to control groups (13.80 ± 3.03; Fig. 2E). The one way ANOVA, confirmed these differences were significant [*F*(2,14) = 195.45, *p* < 0.001]. The post-hoc analysis showed a significant difference between control and MD lesion groups (*t* = 17.18, *p* < 0.001) and between MD sham and MD lesion groups (*t* = 17.06, *p* < 0.001), but did not show a significant difference between control and MD sham groups (*t* = 0.12, *p* > 0.05). During learning phase, the MD lesion group made more errors (median = 5) compared to MD sham (median = 2) and to control groups (median = 2. Fig. 2F). The Kruskal–Wallis one way ANOVA, confirmed these differences [*H*(2) = 9.6, *p* < 0.001]. The post-hoc analysis showed a significant difference between control and MD lesion groups (*q* = 3.97, *p* < 0.001) and between MD sham and MD lesion groups (*q* = 4.98, *p* < 0.001), but did not show a significant difference between control and MD sham groups (*q* = 0.90, *p* > 0.05).

#### 3.3.2 Open field test

**Fig. 3:**
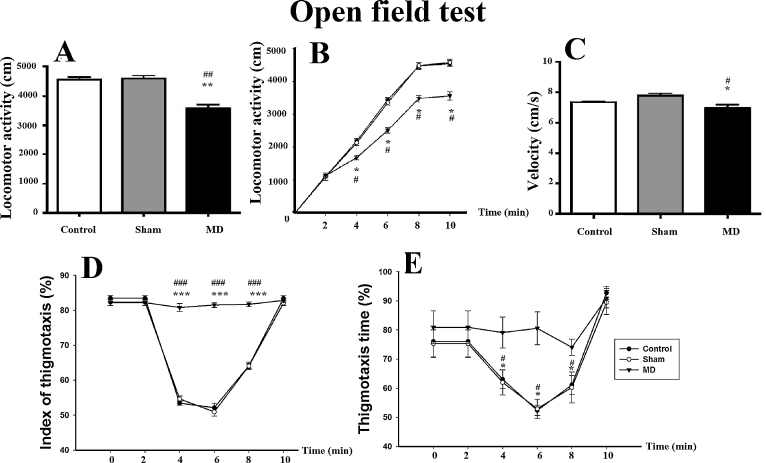
(A–F) Assessment of the behavioral changes in the open field test of adult rats following MD lesion at postnatal day 4 (P4). (A) Mean (±SD) locomotor activity; (B) locomotor activity profile over 10  min; (C) animal’s velocity; (D) assessment of the thigmotaxis patterns during 10–min in the open-field; and (E) thigmotaxis time. The index of thigmotaxis and thigmotaxis time were measured during 5 consecutive periods of 2 min. Mean (±SD). * and #: *p* < 0.05; ** and ##: *p*  < 0.01, *** and ###: *p* < 0.001. The “*” refers to the control vs MD lesion group comparison and the “#” refers to the MD sham vs MD lesion group comparison.

The distance (cm) that the animals moved around the open field during the duration of the test (10 min) was the main dependent measure. The MD lesion animals travelled a smaller distance (3580.92 ± 458.15 cm) in the open field than the MD sham lesion group (4593.61 ± 380.95 cm) and the anesthetized control group (4562.01 ± 306.66 cm; Fig. 3A). Kruskal–Wallis one-way ANOVA of distance travelled confirmed a significant difference between the three groups, [*H*(2) = 21.31, *p *< 0.001]. The post-hoc analysis showed a significant difference between the controland MD lesion groups (*q* = 8.35, *p *< 0.05) and between MD sham and MD lesion groups (*q* = 5.71, *p *< 0.05) but it did not show a significant difference between the MD sham and control groups (*q* = 0.16, *p *> 0.05). We also studied the profile of this locomotor activity (Fig. 3B) by dividing the 10 min of the testing session into 2 min bins. During the first 2 min, the three groups showed the same pattern of locomotor activity: control group (1130 ± 10 cm), MD sham group (1120 ± 112 cm), and MD lesion group (1153 ± 50 cm) [*F*(2,41) = 0.774, *p* > 0.05]. At the following 2 min, locomotor activity of MD lesion animals’ decreased (1703 ± 72 cm) compared to control (2205 ± 75 cm) and to MD sham groups (2150 ± 85 cm; Fig. 3B). One way ANOVA in the mean locomotor activity between the three groups was significant [*F*(2,41) = 175.55, *p* < 0.001]. The post-hoc analysis showed a significant difference between control and MD groups (*t* = 17.08, *p* < 0.001) and between MD sham and MD lesion groups (*t* = 15.21, *p* < 0.001), but did not show a significant difference between the control and MD sham animals (*t* = 1.87, *p* > 0.05). The locomotor activity of MD lesion group decreased significantly in all the following blocks of the test session [*H*(2) = 21.31, *p *< 0.001].

The velocity (cm/s) of the three different lesion groups was also measured during the test session (Fig. 3C). The speed of the MD lesion animals (6.98 ± 0.21 cm/s) was decreased compared to that of the MD sham lesion group (7.78 ± 0.14 cm/s) and the control group (7.35 ± 0.07 cm/s). Kruskal–Wallis one-way ANOVA confirmed this difference was significant, [*H*(2) = 8.27, *p *= 0.016]. The post-hoc analysis showed a significant difference between control and MD lesion groups (*q* = 3.86, *p *< 0.05) and between MD sham and MD lesion groups (*q* = 4.01, *p *< 0.05), but it did not show a significant difference between the MD sham and control groups (*q* = 2.11, *p *> 0.05).

A further assessment of thigmotaxis levels indicated different patterns of this behavior both within the test duration and between the different groups (Fig. 3D). The index of thigmotaxis was calculated as the ratio of the distance covered within the area that is less than 2.5 cm away from the walls compared to the total distance covered, expressed as percentages. The 10 min of the testing session was divided into blocks of 2 min each (i.e., 2, 4, 6, 8, and 10 min). During the first block of 2 min, all three groups showed high levels of thigmotaxis: Control group (83.46 ± 0.7%), MD sham group (82.36 ± 0.96%) and MD lesion group (82.21 ± 0.85%; Fig. 3D). A one-way ANOVA showed no differences between the three groups during this first 2 min of activity in the open field test, [*F*(2,41) = 0.62, *p *> 0.05]. During the second block of 2 min (i.e., between 2 and 4 min), control and MD sham groups started visiting the center of the open-field. Thus their index of thigmotaxis decreased compared to their levels during the first block of 2 min of the test: control group (53.42 ± 0.62%) and MD sham group (54.64 ± 0.71%). However, the thigmotaxis index remained high for MD lesion rats (80.80 ± 1.11%; Fig. 3D) as their behavior indicated that they continued to favor the area adjacent to the outer walls of the box. One way ANOVA of the difference in the mean index of thigmotaxis between the three groups was significant, [*F*(2,41) = 310.55, *p *< 0.001].The post-hoc analysis showed a significant difference between control and MD lesion groups (*q* = 31.19, *p *< 0.001) and between MD sham and MD lesion groups (*q* = 29.80, *p *< 0.001), but it did not show a significant difference between the MD sham and control groups (*q* = 1.38, *p *> 0.05). The constant high index of thigmotaxis in the MD lesion group was maintained throughout all of the test duration. Interestingly, after the fourth block (i.e., after the eighth minute), the index of thigmotaxis started to increase again for the control and MD sham groups. By the end of the test at 10 min, there was no difference in the index for the control group (83.42 ± 0.74%), MD sham group (82.07 ± 0.68%) and MD lesion group (82.85 ± 0.60%; Fig. 6D), [*F*(2,41) = 1.00, *p *> 0.05].

Concerning the thigmotaxis time (Fig. 6E), during the first block of 2 min, all the three groups spent more time close to the apparatus walls: control group (76.06 ± 5.25%), MD sham group (75.34 ± 4.38%) and MD lesion group (80.94 ± 5.67%; Fig. 6E). A one way ANOVA showed no differences between the three groups during this first 2 min of activity, [*F*(2,41) = 2.71, *p* > 0.05]. In the course of the second block of 2 min, control and MD sham groups spent less time near the walls and started visiting the center of the apparatus. Thus, their thigmotaxis time decreased compared to their levels over the first blocks: control group (63 ± 3.2%), MD sham (62 ± 4.36%). However, the thigmotaxis time for MD lesion group remained high (79.13 ± 5.33%; Fig. 6E). Kruskal–Wallis ANOVA of the difference between the three groups was significant, [*H*(2) = 27.73, *p* < 0.001]. The post-hoc analysis showed a significant difference between control and MD lesion groups (*q* = 9.08, *p *< 0.05) and between MD sham and MD lesion groups (*q* = 9.08, *p* < 0.05), but did not show a significant difference between control and sham groups (*q* = 0.94, *p *˃ > 0.05). By the end of the test, the thigmotaxis time started to increase again for the control and MD sham groups and at the last block of 2 min, there was no significant difference in thigmotaxis time for control group (92.78 ± 2.19%), MD sham group (89.5 ± 4.1%) and MD lesion group (90.92 ± 3.36%; Fig. 6F), [*F*(2,41) = 3.47, *p *> 0.05].

#### 3.3.3 Actimetry

**Fig. 4:**
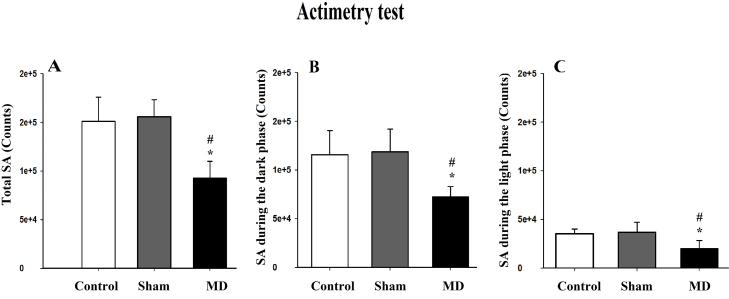
(A–C) Assessment of spontaneous activity by Actimetry: (A) mean (±SD) total spontaneous activity. (B) Mean (±SD) spontaneous activity during the dark phase and (C) mean (±SD) spontaneous activity during the light phase. * and #: *p*  < 0.05. The “*” refers to the control vs MD lesion group comparison and the “#” refers to the MD sham vs MD lesion group comparison.

Animals were exposed to an identical lighting cycle to assess the effects of MD lesion on locomotor abilities; data acquisition was computerized and analyzed automatically. The activity records were expressed by counts (activity per minute). The total spontaneous activity (SA) was modified after the MD lesion (Fig. 4). MD lesion rats showed a significant reduction in theiractivity (92,574.20 ± 17,367.08 counts) compared to MD sham (155,518.80 ± 17,693.4 counts) and control rats (156,875.8 ± 25,101.27 counts; Fig. 7A). The one way ANOVA confirmed that this difference was significant [*F*(2,14) = 14.82, *p *< 0.001]. The post-hoc analysis showed a significant difference between the control and MD lesion groups (*t* = 4.25, *p *< 0.01) and between MD sham and MD lesion groups (*t* = 4.88, *p* < 0.001), but did not show a significant difference between the control and MD sham groups (*t* = 0.36, *p* > 0.05).

The SA over the dark period was also affected by MD lesion. The MD lesion group showed a decreased activity (72,446 ± 10,432 counts) compared to MD sham (118,698 ± 23,217.70 counts) and controls (115,676.80 ± 24,690.72 counts; Fig. 4B). The Kruskal–Wallis one way ANOVA confirmed this difference was significant, [*H*(2) = 7.76, *p *< 0.05].

The SA over the light period was also reduced by MD lesion (Fig. 7C). The MD lesion group exhibited less activity (20,005.4 ± 8234.48 counts) compared to MD sham (36,826.8 ± 10,207.01 counts) and control groups (35,199 ± 4808.72 counts; Fig. 4C), the one way ANOVA confirmed this decrease in activity [*F*(2,14) = 6.61, *p * < 0.05]. The post-hoc analysis showed a significant difference between control and MD lesion groups (*t* = 2.97, *p* < 0.05) and between MD sham and MD lesion groups (*t* = 3.29, *p *< 0.05), but did not show a significant difference between control and MD sham groups (*t* = 0.31, *p* > 0.05).

#### 3.3.4 Elevated plus maze

**Fig. 5:**
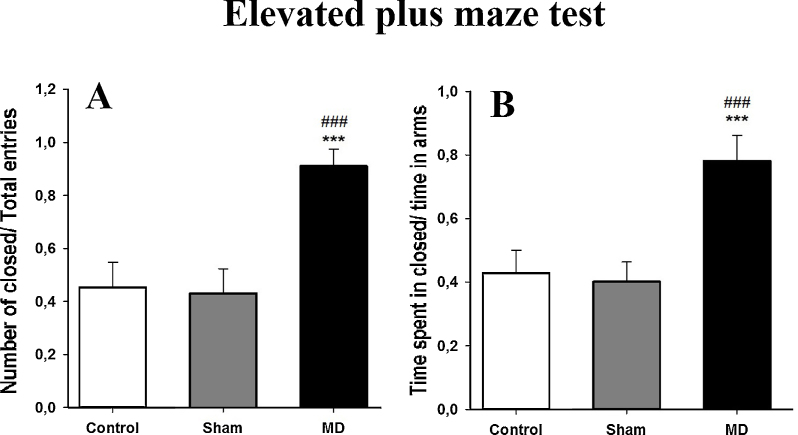
(A and B) Evaluation of anxiety in the elevated plus maze test: (A) mean (±SD) ratio (*R*) of closed arms number of entries over the total entries made into each area. (B) Mean (±SEM) ratio (*R*) between time spent in the closed arms relative to time spent in the open arms. *** and ###: *p* < 0.001. The “*” refers to the control vs MD lesion group comparison and the “#” refers to the MD sham vs MD lesion group comparison.

During this test, the critical measure is the amount of time that the animal explores the closed arms relative to the total amount of time they explore the open and closed arms of the maze, expressed as a ratio (*R*). The MD lesion group spent more time in the closed arms, the ratio was (0.78 ± 0.08) compared to the MD sham (0.40 ± 0.06) and the control groups (0.43 ± 0.07; Fig. 5B). One-way ANOVA confirmed that this difference in exploration times was significant, [*F*(2,14) = 2122.70, *p *< 0.001]. The post-hoc analysis showed a significant difference between control and MD lesion groups (*t* = 14.05, *p *< 0.001) and between MD sham and MD lesion groups (*t* = 13.02, *p* < 0.001) but it did not show a significant difference between the MD sham and control groups (*t* = 1.03, *p *> 0.05).

The total number of arm entries made into each of the two types of arms was also assessed, expressed as a ratio of the total number of closed arm entries divided by the total number of entries into both open and closed arms. The MD lesion rats visited the closed arms more frequently (*R* = 0.91 ± 0.04) compared to the MD sham rats (*R* = 0.43 ± 0.09) and the control rats (*R* = 0.45 ± 0.09; Fig. 5A). One-way ANOVA confirmed that this difference in the mean number of entries was significant, [*F*(2,14) = 142.70, *p* < 0.001]. The post-hoc analysis showed a significant difference between control and MD lesion groups (*t* = 14.94, *p *< 0.001) and between MD sham and MD lesion groups (*t* = 14.22, *p *< 0.001) but it did not show a significant difference between the MD sham and control groups (*t* = 0.77, *p *> 0.05).

#### 3.3.5 Social contact test

**Fig. 6:**
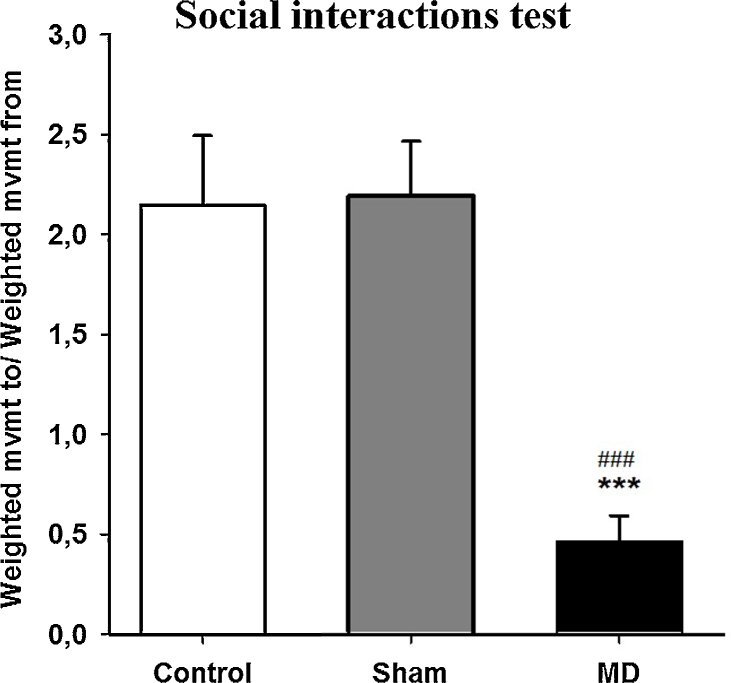
Social contact test showing the mean (±SD) ratio between the weighted movement to and the weighted movement from the paired rat. *** and ###: *p* < 0.001. The “*” refers to the control vs MD lesion group comparison and the “#” refers to the MD sham vs MD lesion group comparison.

This test measured the levels of social anxiety in the open field based on the animals’ interactions. The interactions consisted of two sequences: the recognition by sniffing, and animal intensity approaches and avoidance. As mentioned previously, the ratio (*R*) between “the weighted movement to” and “the weighted movement from” was evaluated. The behavioral analysis showed that the MD Sham animals approached the Control animals, whereas the MD lesion animals avoided them (Fig. 6). The statistical analysis of the intensity of ratio (*R*) showed that MD lesion animals have a tendency to avoid the stimulus rat (0.46 ± 0.13) compared to the MD sham animals (2.19 ± 0.27) and to the control (2.14 ± 0.35). Kruskal–Wallis one-way ANOVA confirmed that this difference in the intensity of animals’ approaches and avoidance is significant [*H*(2) = 27.49, *p *< 0.001]. The post-hoc analysis showed a significant difference between control and MD lesion groups (*q* = 9.52, *p *< 0.05) and between MD sham and MD lesion groups (*q* = 6.42, *p *< 0.05) but it did not show a significant difference between the MD sham and control groups (*q* = 0.06, *p *> 0.05).

#### 3.3.6 Passive avoidance test

Rats tested in the passive avoidance paradigm showed no substantial increase in median response latency during the first two testing sessions (MD lesion (2 ± 0.23 s); MD Sham (2 ± 0.19 s); control (2 ± 0.25 s)). During the third testing session, the avoidance latencies started increasing in MD sham (100.5 ± 4.34 s) and control rats (80.75 ± 2.0865 s), but they remained lower in MD lesion rats (2 ± 0.25 s). During the fourth testing session, there was a marked increase in avoidance latency displayed in MD sham (360 ± 0 s) and control rats (360 ± 0 s), but not in MD lesion rats (60 ± 1.12 s). However, latencies to respond did increase further for MD lesion rats on subsequent training sessions and reached 360 s on day 6.

**Fig. 7:**
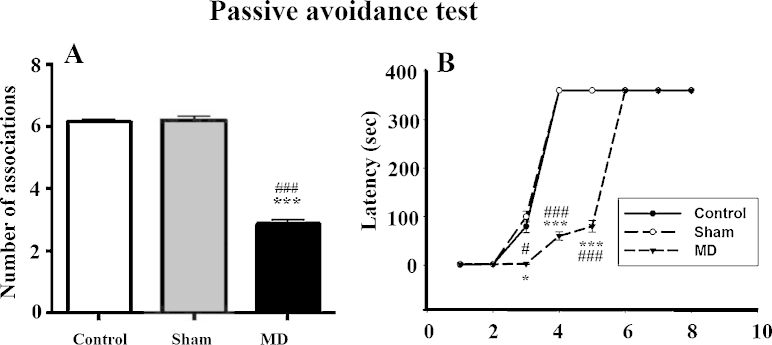
(A and B) Assessment of the MD lesion effects on cognition using the passive avoidance test: (A) mean (±SEM) number of associations and (B) latencies (s) during the eight different test sessions. * and #: *p* < 0.05; *** and ###: *p*  < 0.001. The “*” refers to the control vs MD lesion group comparison and the “#” refers to the MD sham vs MD lesion group comparison.

The total number of associations (avoidance responses) made by each group indicated a deficit in learning for the MD lesion group, who recorded fewer associations (2.86 ± 0.53) compared to MD sham animals (6.21 ± 0.43) and control group (6.14 ± 0.36). Kruskal–Wallis one-way ANOVA analysis confirmed that the difference in the number of associations was significant [*H*(2) = 33.38, *p *< 0.001; Fig. 7A,B]. The post-hoc analysis showed a significant difference between control and MD lesion groups (*q* = 9.32, *p *< 0.05) and between MD sham and MD lesion groups (*q* = 6.56, *p *< 0.05) but it did not show a significant difference between the MD sham and control groups (*q* = 0.45, *p *> 0.05).

## 4 Discussion

The present series of experiments sort to determine if there were changes to behavior and cognition in adult rats as a consequence of early postnatal MD lesions timed at day P4 after birth. These experiments have provided several main findings to support our hypothesis that early onset damage sustained to the MD will cause behavioral and cognitive deficits. The morphological and behavioral effects of the lesion on the development of the PFC indicate that after early MD lesions, a significant decrease of pyramidal cell density was observed within the cingulate cortex and the dorsolateral and infralimbic subdivisions of the PFC. Adult rats exhibited impairments in their ability to learn the spatial delayed alteration test, which is known to depend upon the integrity of the PFC [37]. MD lesion rats had, during adulthood, high measures of anxiety-like behavior, deficits in social behavior and learning, and changes in locomotor activity. Specifically, following MD lesion at P4, we found that when the rats were tested in adulthood, their locomotor activity was decreased and they displayed increased levels of thigmotaxis, but there was no change in their velocity. Further, they did not engage in as many social interactions and they displayed more anxiety-like behaviors in the elevated plus maze and open field tests compared to control and MD sham lesion rats. The early MD lesions also reduced the numbers of learned associations and produced delays in acquisition during the passive avoidance test. The present data demonstrate that damage to MD in the first postnatal week results in long term behavioral and cognitive abnormalities in adult rats, presumably by disrupting circuitry forming between the MD and PFC during this critical period in brain maturation [25,38].

Previous rodent studies, e.g., the behavioral study of Floresco and Grace [39] have suggested that afferents from MD into the medial PFC may modulate the activity of interactions between the medial PFC-hippocampal neurons that are essential components of cortical networks regulating information processing. Our current results, after damage to the MD at P4, have produced different results to other studies (e.g., [23,40]) that caused damage to PFC or MD when the rats were adults. Thus, our current results provide important insights into the consequences of changes in thalamo–cortical interactions during brain maturation and their impact on normal cognitive and behavioral functioning in adulthood as discussed below.

Given that the behavioral and cognitive effects were only present in the MD lesion group and not in the MD Sham lesion or anesthetized control groups, it may be assumed that any effects of the procedures or anesthetic methods [23,40] are minimal and that the behavioral and cognitive deficits are the consequences of changes induced to the MD at such an early age during brain maturation. The early postnatal period (from P1 to P10) in the rat brain is characterized by dramatic changes contributing to synaptic formation, growth, regression and stabilization of connections [41]. As previously mentioned, neurosurgery was conducted on day P4, to coincide within the range of time when afferent and efferent connections are forming between the MD and PFC [24]. Interestingly, Van Eden [24] suggested that MD projections are shaping the PFC as demonstrated by thalamo–cortical afferents arriving at the cortex in layer III before the cortico–thalamic afferents arrive within the thalamus (typically by P4 and P5). Further, the MD has many neurons at birth that begin to reduce extensively by postnatal day P13 [24]. Data from other studies on brain maturation in rodents suggests that manipulations that occur to the neuronal structures prior to postnatal day 7 (P7) produce permanent changes in the brain that persist into adulthood. In contrast, manipulations occurring on or after P7 do not cause similar long-term changes in the brain, suggesting that these later changes have little or no effect on the development of the cortex [38].

Our MD lesions produced on P4 were performed using electrolytic heating. This method damages cell bodies as well as axons passing through the lesion site. Consequently, while our lesions are confined to the MD (with some damage to the paraventricular nucleus located in the midline of the thalamus), there will also be additional non-specific damage to fibers of passage coursing through our lesion site. Therefore, the cognitive and behavioral data observed in the MD lesion rats compared to the two control groups may be attributed to dysfunction in cortico–thalamo–cortical projections associated with the MD, in addition to other structures as well. For example, it cannot be ruled out that the early lesion caused at P4 may have also disrupted the critical connections being formed between paraventricular thalamic nucleus and its interconnected regions of the brain as well [42].

Our histology also revealed some neuronal atrophy in the hippocampus (Fig. 1). This shrinkage of the hippocampus could be explained by the data from the study of Floresco and Grace [39], which showed that afferents from MD modulate the activity of PFC-hippocampus neurons. Developmental studies estimated that in rats, 70% of granular cells are produced during the first two postnatal weeks [43], and that in CA3, the density of glutamatergic receptors doubles from P0 to P15. The MD receives direct projections from the subiculum and indirect hippocampal projections via a number of limbic structures, including the amygdala and entorhinal cortex [43]. The MD projects densely to various layers of the PFC [44,45]. Thus it is conceivable that these alterations in the hippocampus depend upon the presence of PFC-hippocampal neurons developing abnormally in the context of the loss of the MD inputs. It may also be suggested that our lesion, which caused damage within the midline thalamic region, may have also disrupted the formation of connections between these thalamic structures, the limbic cortex and extended hippocampal formation [42,46,47]. Further studies are required to be able to assess more widespread changes in these neural networks as a consequence of early onset damage to the MD.

Consequently, the evidence of neuronal changes in these regions as a consequence of bilateral damage to the MD and adjacent thalamic nuclei produced at day P4 are important to elucidate given the clear behavioral and cognitive deficits observed in these adult rats. Our results showed that the early onset MD lesion caused a significant decrease in pyramidal cell density in comparison to sham and controls. These findings are consistent with previous results showing significant decreases in pyramidal cell density within the PFC after MD lesion performed at P0 [25].

Changes in cognition were assessed using several different tasks. The spatial delayed alternation behavior was studied to reveal whether or not the absence of major thalamic inputs into the PFC leads to a disorganization and malfunction of the PFC. One of the key findings in the present study was the deficit in learning to switch a response in both tasks. During the nonmatching-to-place task, MD lesion rats performed a small percentage of correct choices during the four blocks of acquisition and showed a very strong perseverative behavior. In both perseveration and learning phases, the MD lesion group made greater mean errors compared to control and sham rats.

These results echo the deficits produced in rats with PFC damage [48–52]. The MD lesioned animals exhibited a perseverative side bias behavior; during all the task sessions they visited more frequently the right arm compared to the left. Dias and Aggleton [53] suggested that the deficit in nonmatching-to-place may influence animal’s performance on the matching-to-place and reversal tasks, and thus may facilitate their acquisition accidentally. To exclude this bias effect, during the matching-to-place, we associated the reward to the less visited arm. On initial acquisition trials, all groups including controls, performed significantly below chance. MD lesioned rats showed excessive perseveration to visit the right arm of the maze; this side bias made learning the matching rule more difficult. We showed that the early MD-lesion impaired the acquisition, but not the performance compared to sham and control rats. These findings are supported by the study of Hunt and Aggleton [50]. By dividing acquisition into two phases, “Perseveration” and “Learning”, it was found that perseverative errors were significantly greater in MD lesioned animals compared to sham and controls. The distinctions between the perseverative and learning phases are interesting as they provide a direct analytical comparison that distinguishes the effect of our lesion on both matching- and nonmatching-to-place tasks.

The surprising finding is that MD-lesioned animals exhibit a combined phenotype of behavior; they presented not only the same deficits reported previously after adult MD lesion (see [50]) but echo also the deficits produced in rats with large mPFC damage [49,54]. It is possible that such multiple effects could interact with one another to compound or enhance existing effects, or result in a new effect altogether. The present cognitive study, therefore, indicates that studying the contributions made by MD afferents are also important for advancing our fundamental understanding of PFC function. We have showed that aspects of response, control and inhibition are altered after early MD lesions causing disruption of egocentric spatial working memory.

Specifically, early MD lesions lead to a selective deficit in the ability to switch from a preferred strategy to a new strategy. The initial acquisition deficit arose from a failure to switch from an innately preferred strategy (nonmatching) to a new strategy (matching), as reflected by a specific increase in perseverative errors. Similarly, the abnormal pattern of errors in the subsequent reversal to a nonmatching rule also arose from an excess of perseverative errors [55]. This would also explain the deficits found in the radial arm maze, when the selection of some arms is never rewarded [56], because the rat now has to withhold a foraging strategy of visiting all arms.

In the present study, we also investigated if an early MD lesion may produce, in adulthood, a variety of behavioral abnormalities reminiscent of schizophrenia. Our data in the open field test showed that the distance traveled by MD lesion rats was decreased compared to the MD sham and control groups, although their speed was not affected. Our results are in contrast to another study that reported no difference in distance traveled in adult rats with MD damage sustained at postnatal day 7 [23]. Our results also contrast with other studies that reported mice and rats with MD lesions received during adulthood showed higher levels of movement in the open field test [50,57]. On reflection, our results may appear to be confounded by some effect of anxiety-like behavior in the MD lesion group. However, the assessment of spontaneous activity confirmed these results and thus, we suggest that other explanations should also be considered. For example, our evidence of reduced locomotor activity after P4 neonatal MD lesions is in contrast to other studies that reported higher levels of anxiety to be positively correlated with an increase in locomotor activity after neonatal lesions of the ventral hippocampus [58–60].

Consequently, these different patterns of locomotor activity may be due to differences in the loci of the thalamic lesions. For example, MD lesions can also affect the adjacent anterior thalamus (ATN) as was reported to have occurred in the rats of Hunt and Aggleton [56]. Interestingly, the endocrine response to stress is affected in rats with damage to the ATN [61]. Thus, adult MD lesions combined with ATN damage may show increased locomotor activity. However, our MD lesion rats with reduced locomotor activity also had some damage to the ATN, although it was small and limited to the anterodorsal subdivision of ATN. In contrast, the MD lesions in Lipska et al. [23] were reported to be smaller, and their photomicrograph indicates that some neurons in MD have been spared (see their Fig. 2E). Additionally, the study of Suarez et al. [62] showed that neonatally (P7) MD lesion rats showed reduced vertical activity in response to amphetamine injections and no changes in locomotor activity to novelty after saline or amphetamine injections 7 weeks post-lesion. Further, their adult MD lesion rats exhibited no changes in motor activity when compared to controls at 7 weeks post-lesion. Thus they concluded that neonatal or adult excitotoxic lesions of MD do not produce behavioral changes analogous to those seen after neonatal ventral hippocampal lesions and do not appear to reproduce animal model-like features of schizophrenia [63]. Our evidence of reduced locomotor activity in adult rats with MD lesion produced at P4 is therefore important for two reasons. Firstly, the lesions were produced during the timeframe identified by Kolb and Cioe [64] as being critical to ensure long-term changes in the brain. Secondly, the reduced locomotor activity is likely to be a consequence of these long-term changes in the brain produced by disruption to the MD at this early stage of brain development. Thus, our evidence for reduced locomotor activity may be the result of higher levels of anxiety-like behavior (as evidenced by the extensive thigmotaxis observed in these MD lesion rats as discussed below). However, if this is so, then changes in anxiety-like behaviors as measured by locomotor activity presents differently after MD lesions at P4 when compared to MD lesions in adulthood and when compared to neonatal and adult lesions of ventral hippocampus [23,59].

Interestingly, the results of thigmotaxis identified that three distinct epochs of exploration appeared to exist during the 10 min of the open field test that distinguished behavior between the three groups. Importantly, the index of thigmotaxis calculated in the open field test was recorded and collected using video-tracking technology to ensure precise and objective measures. During the first 2 min of the test, all three groups displayed constant thigmotaxis, which may reflect the animals’ natural curiosity to develop a spatial representation of the boundaries of their environment [65]. Secondly, after this initial thigmotaxis exploration, the MD sham and control group rats explored the entire open field box during the subsequent 6 min of the test. In contrast, the MD lesion rats continued to explore the outer areas of the box closest to the walls, so consequently continued to display thigmotaxis exploration. Thirdly, after 8 min in the open field test, the animals’ motivation for exploration appeared to decrease with sham MD, control, and MD-lesion group rats reducing their movements or sleeping periodically in different locations of the open field environment; thus the thigmotaxis measure does not appear as reliable. However, as the results indicate the differences in the high levels of thigmotaxis that were apparent throughout the test in the MD lesion group, but not in the MD sham and control groups suggests that the MD lesion group displayed more anxiety-like behavior. A marked increase of thigmotaxis in rodents has been previously correlated with aggravation of anxiety levels and also with anxiogenic drugs, including amphetamine [66], pentylenetetrazole [67] and idazoxan [68].

After MD lesions at postnatal P4, our adult rats produced more anxiety-like behavior during the elevated-plus maze test compared to MD sham lesion and control groups. Similar results have been reported in mice with MD created in adulthood [14,69], suggesting that the anxiety-like behavior that is observed during this elevated-plus maze task is not necessarily developmental driven. Notably, the elevated plus maze test involves a motivational conflict – the rats’ innate tendency to explore a novel environment is opposed to their innate fear of open spaces [40]. Our data observed in these two tests (open field and elevated-plus maze) provide unique insight into the differences in anxiety-like behavior of adult rats with MD damage sustained at P4. As observed, MD lesion rats display less movement in the open field test in contrast to higher locomotor activity in rats with neonatal ventral hippocampal lesions or no change in locomotor activity after adult MD lesions [40,41,63], and they are more likely to seek out the closed arm spaces in the elevated-plus maze task compared to the MD sham lesion and control groups. Anxiety is associated with heightened distractibility, poor concentration and an increased responsiveness to potential threat [70]. Neuroimaging evidence demonstrates a link between anxiety-like behaviors and the medial PFC in humans [69], although there are mixed results on measures of anxiety-like behavior in rat models after medial PFC lesions [70,71]. Consequently, our novel evidence of changes to levels of anxiety-like behaviors after early onset damage to the MD suggests that the interactions between the MD and PFC are important in anxiety-like behaviors.

Our study also reports for the first time, the effects of early MD lesions in rodents performing a social contact test. To our knowledge, only one other study has assessed the MD involvement in social interaction using adult rats [19]. Our findings demonstrated that the P4 MD lesion caused an alteration in social interactions during adulthood, whereby these animals avoided the stimulus rat more than MD sham and control animals. Interestingly lesions to the PFC at differing stages of development in rodents have reported contrasting effects. Dopaminergic lesions of the medial PFC during adolescence caused an extensive impairment in socially interactive behavior [72,73] while medial PFC lesion adult rats spent significantly longer periods of time in social interactions [74], suggesting an anxiolytic-like effect on behavior due to the damage to the medial PFC in adulthood. Our data suggest that the behavioral effects linked to early onset damage during brain maturation in the MD is comparable to damage in the medial PFC sustained during adolescence [72] in leading to impaired social interactions during adulthood. Several studies report that people with social phobia experience the full range of symptoms typically associated with anxiety [75]. These reports accord well with our data, as our P4 MD lesion adult rats demonstrated less interest in social interactions with other rats, as well as heightened levels of anxiety-like behavior on the elevated-plus maze and increased levels of thigmotaxis despite decreased locomotor activity in the open field test.

Finally, the passive avoidance test showed that our adult rats with MD lesions produced at P4 were slower at acquiring the associations compared to the MD sham and control groups. In addition, the MD lesions affected the number of learned associations. Further, our results indicate that an intact MD thalamus is important during the initial stages of learning in this task. In our study, the consistently higher differences in latency measures observed during the earlier testing sessions between the MD lesion and the control groups raises the possibility of a generalized deficit that affected learning in this task. However, after subsequent repeated exposures to the same testing conditions, rats with MD lesions were able to acquire the associations and began to avoid entering the darkened chamber and avoid shock. Previous work [50] has demonstrated that adult rodents with MD lesions also have learning deficits in the passive avoidance task. In contrast, medial PFC lesions do not affect the acquisition of two-way avoidance [72] nor alter the acquisition of the passive avoidance test [76]. During the initial sessions when the animals were learning the associations of the passive avoidance task, rats with MD lesions appeared unable to decide to avoid the goal-box by recalling the specific shock experience. In other behavioral paradigms, rodents, primates and humans with MD lesions sustained during adulthood are impaired at adaptive decision-making and associative recognition memory tasks [1,6,11,16–18]. However, the deficits in learning these associations cannot be attributed to higher levels of anxiety in general, and our P4 MD lesion rats were slowly able to acquire the association and avoid the compartment with the electric shock. In other rodent studies, damage to the MD during adulthood has been reported to cause an inability to adopt different strategies, or withhold spatial responses [77], or impair working memory in adult rats [78]. All of this evidence after MD lesion damage in rodent models has been attributed to impaired executive functioning and changes in cognitive control. Interestingly though, as mentioned above, early postnatal damage to MD caused by electrolytic lesions does not impair learning operant delay alternation in rodents [25] and adult MD lesion damage leaves learning intact for this task as well [14]. Consequently, our behavioral and cognitive evidence suggests that damage to the MD at day P4 produces widespread alterations in several neural networks important for specific forms of cognition and behavior. Importantly, these deficits are not generalizable to all behavioral responses typically attributed to the PFC, and the MD lesion rats are able to learn specific associations over time.

Elucidating mechanisms that underlie the effects of MD lesions at P4 will require further studies on the anatomical and molecular changes that take place between the lesion time and the emergence of deficits. Previous developmental studies have shown that the loss of MD cells at an early stage of brain maturation leads to a decrease in spine density [38]. The study of Marmolejo et al. [21] revealed a decrease in the expression of certain proteins involved in the stabilization of dendritic formations, which may be responsible for this imbalance. Jones et al. [38] also noted that changes in spine densities are observed in patients with schizophrenia. Others have proposed that the underlying mechanisms in developmental disorders like schizophrenia may be due to alterations in optimal thalamo–cortical interconnections, which lead to an imbalance of excitatory and inhibitory communication through altered neural circuitry [43]. According to our model, early MD lesions sustained at day P4, led in early adulthood to the emergence of abnormalities in a number of dopamine-related behaviors [79,80], which bear close resemblance to behaviors seen in animals sensitized to psycho-stimulants [78–83] including high levels of anxiety-like behaviors, impaired social behaviors, learning and working memory problems and stereotypic behaviors. Many of these cognitive and behavioral phenomena show parallels with schizophrenia.

To conclude, these results suggest that the functional contribution of the MD is important for supporting optimal cortico–thalamo–cortical interactions from very early on during brain maturation. The thalamus is the primary excitatory input to prefrontal cortical regions. Our evidence indicates that it is critically important to better understand the functional roles of MD (as well as other adjacent thalamic nuclei) in the development of the PFC, in order to understand how the cortex is involved in cognition. Further, advancing our understanding of these cortico–thalamo–cortical interactions may provide potential targets for more effective treatments in many neuropsychiatric disorders, like schizophrenia.

## Conflict of interests

None declared.
