# Supplementary material for: Behavioral and cognitive changes after early postnatal lesions of the rat mediodorsal thalamus
Source: Behav Brain Res. 2015 Oct 1;292:219–32. doi: 10.1016/j.bbr.2015.06.017 (PMC4571833; doi:10.1016/j.bbr.2015.06.017)
Supplement: Supplementary file 1 [file mmc1.doc]

**Figure legends**

**Figure 1:** Photomicrographs detailing the extent of the damage to the MD at P4**(A),** inadulthood rats which had bilateral lesions performed at P4 (**B),** and also the extent of the damage to the MD and to the ATN in adult rats**(C)**. Numbers represent distance from bregma. **D**) Assessment of early MD lesion at postnatal day 4 (P4) effect on pyramidal cells development in the prefrontal cortex. ** and ##: p˂ 0.01. The “*” refers to the control vs MD lesion group comparison and the “#” refers to the MD sham vs MD lesion group comparison.

**Figure2:** Acquisition of the delayed alternation task in T-maze. **A-C**: Animals performance in a nonmatching-to-place task. **A**: The pattern of acquisition of the T-maze nonmatching-to-place over four blocks of three sessions. **B-C**: Mean (± SD) group error scores have been divided into two phases: Perseveration phase (**B**) in which rats are performing below chance and learning phase (C) in which rats are performing at or above chance. **D-F**: Acquisition of the delayed alternation responses in T-maze matching- to-place task caused by MD lesion at postnatal day 4 (P4). **D**: The pattern of acquisition of the T-maze matching-to-place over six blocks of 30 trials sessions. **E-F**: Mean (± SD) group error scores have been divided into two phases: Perseveration phase (**E**) in which rats are performing below chance; learning phase (**F**) in which rats are performing at or above chance. * and #: p<0.05; ***: p˂ 0.001. The “*” refers to the control vs MD lesion group comparison and the “#” refers to the MD sham vs MD lesion group comparison.

**Figure 3:A - F**: Assessment of the behavioral changes in the open field test of adult rats following MD lesion at postnatal day 4 (P4).**A**: Mean (± SD) locomotor activity; **B**: Locomotor activity profile over 10 min; **C**: animal’s velocity; **D**: Assessment of the thigmotaxis patterns during 10 min in the open-field; and **E**: thigmotaxis time. The index of thigmotaxis and thigmotaxis time were measured during 5 consecutive periods of 2 min. Mean (±SD). * and # p<0.05; ** and ##: p˂ 0.01, *** and ###: p<0.001. The “*” refers to the control vs MD lesion group comparison and the “#” refers to the MD sham vs MD lesion group comparison.

**Figure 4: A**- **C**: Assessment of spontaneous activity by Actimetry: **A**: Mean (± SD) total spontaneous activity. **B**: Mean (± SD) spontaneous activity during the dark phase and **C**: Mean (± SD) spontaneous activity during the light phase. * and #: p˂ 0.05. The “*” refers to the control vs MD lesion group comparison and the “#” refers to the MD sham vs MD lesion group comparison.

**Figure 5: A – B**: Evaluation of anxiety in the elevated plus maze test: **A**: Mean (± SD) ratio (R) of closed arms number of entries over the total entries made into each area. **B**: Mean (± SEM) ratio (R) between time spent in the closed arms relative to time spent in the open arms. *** and ###: p˂ 0.001. The “*” refers to the control vs MD lesion group comparison and the “#” refers to the MD sham vs MD lesion group comparison.

**Figure 6:** Social contact test showing the mean (± SD)ratio between the weighted movement to and the weighted movement from the paired rat. *** and ###: p˂ 0.001. The “*” refers to the control vs MD lesion group comparison and the “#” refers to the MD sham vs MD lesion group comparison.

**Figure 7: A – B**: Assessment of the MD lesion effects on cognition using the passive avoidance test: **A**: Mean (± SEM) number of associations and **B**: latencies (s) during the eight different test sessions. * and #: p<0.05; *** and ###: p˂ 0.001. The “*” refers to the control vs MD lesion group comparison and the “#” refers to the MD sham vs MD lesion group comparison.
